# Relationship between perceived stress, stress coping strategies, and clinical status in patients with rheumatoid arthritis

**DOI:** 10.1007/s00296-023-05367-6

**Published:** 2023-06-18

**Authors:** Aldona Wróbel, Ilona Barańska, Joanna Szklarczyk, Anna Majda, Jolanta Jaworek

**Affiliations:** 1 Laboratory of Nursing Theory and Fundamentals, Institute of Nursing and Midwifey, Faculty of Health Sciences, Jagiellonian University Medical College, Michałowskiego 12 Street, 31-126, Krakow, Poland; 2 Laboratory for Research On Aging Society, Department of Sociology of Medicine, The Chair of Epidemiology and Preventive Medicine, Jagiellonian University Medical College, Krakow, Poland; 3 Department of Medical Physiology, Institute of Physiotherapy, Faculty of Health Sciences, Jagiellonian University Medical College, Krakow, Poland

**Keywords:** Rheumatoid arthritis, Coping strategies, Perceived stress, Active disease

## Abstract

Coping with a chronic disease such as rheumatoid arthritis (RA) involves significant changes in life and promotes stressful situations. The inability to cope with stress can contribute to the lack of effectiveness of therapy. The aim of this study was to evaluate the relationship between perceived stress, coping strategies, and the clinical status of RA patients determined by C-reactive protein (CRP) and Disease Activity Score (DAS28). 165 subjects were studied, 84 of them had RA and the rest were controls. Standardised questionnaires were used: the Inventory for the Measurement of Coping Strategies (Mini-COPE) and the Perceived Stress Scale (PSS-10). A self-administered questionnaire was used to collect sociodemographic data. The blood levels of protein CRP and cortisol were determined. DAS28 was obtained from medical records. The study was cross-sectional. The mean severity of perceived stress PSS-10 was not significantly different between the control and study groups. RA patients most often used coping strategies such as active coping, planning, and acceptance. Compared to the control group, they used the strategy of turning to religion significantly more often (1.8 vs 1.4; *p *= 0.012). Women with RA who had higher cortisol levels were more likely to use positive reevaluation, seeking emotional support and instrumental support, as well as the denial strategy. In men with RA, high stress was associated with twice as high CRP levels compared to patients with low stress (*p *= 0.038). As the levels of CRP protein levels (*p *= 0.009) and the DAS28 index (*p *= 0.005) increased, patients were more likely to use a denial strategy.

## Introduction

The experience of chronic disease is one of the stressors that accompany human life. However, not only the illness that can cause stress, but stress that occurs can also contribute to the onset of illness. The inability to cope with stress results in a breakdown in health as a result of the depletion of the body’s immune resources (hormonal, immune). The nervous and immune systems interact with each other and communicate through the sympathetic nervous system–adrenal medulla axis (adrenaline) and the hypothalamic–pituitary–adrenal cortex axis (cortisol). Depending on the duration and intensity of the stressful situation, these hormones determine whether stress has a stimulating or inhibitory effect on the immune system. The experience of severe and prolonged stress inhibits the response of the humoral and cellular immune systems, while a short and less-intense stress stimulates it. Chronic stress and the inability to cope with it result in sustained high blood cortisol concentrations. This situation leads to hyperadaptosis (increased tolerance to stress hormones) and consequently to the onset of disease. The body’s functioning in constant readiness to defend itself against stress leads to depletion of its resources. This condition weakens the immune system and contributes 80–90% to the development of disease [1–7].

Struggling with a chronic somatic disease, such as rheumatoid arthritis (RA), is often associated with an inability to fulfil social roles, giving up some life goals, difficulties in meeting important needs, frequent hospitalisations, and undergoing medical treatments and procedures. These are reasons that can be an additional source of stress for chronic patients [8]. Studies show that the proportion of patients suffering from depression is higher among those diagnosed with rheumatic disease compared to the general population. This has a significant impact on the treatment process for these patients, as patients with additional psychiatric disorders respond less well to somatic treatment. The appearance of RA is perceived by the patient as a source of chronic stress [9]. Coping with stressful situations is regarded as a complex process that changes over time. In patients with chronic illness, it can last for several years. There are, however, many different coping strategies for dealing with stressful situations: confrontation, distancing, self-control, seeking social support, accepting responsibility, avoidance, planned problem solving, and positive re-evaluation. Incompetent coping in a stressful transaction can lead to health disorders, the development of somatic diseases and contribute to the lack of effectiveness of the therapy provided [8, 10–12].

Therefore, the objective of the presented cross-sectional study was to evaluate the relationship between perceived stress, coping strategies, and the clinical status of patients with RA as determined by C-reactive protein (CRP) and Disease Activity Score (DAS28). To test whether stress, understood as a negative experience, can affect the clinical condition of patients, we were the first in Poland to conduct a study among patients with rheumatoid arthritis, which examines stress and its impact on the clinical condition of this group of patients. To strengthen the power of the study, we used standardised survey questionnaires and correlated them with laboratory analysis.

## Methodology

### Study design and setting, group

This cross-sectional survey was conducted in 2018 in the Outpatient Clinic (control group) and the Rheumatology Department (study group) of the hospital in Kraków, Poland. The study group consisted of patients aged 18–70 years with diagnosed RA confirmed by a rheumatologist, without diagnosed mental disorders, who gave their informed and voluntary written consent to participate in the study.

The control group consisted of people between the ages of 18 and 70. They had not diagnosed RA, other serious chronic or autoimmune diseases, or psychiatric disorders. They had no joint pain or swelling on the day of the study. They gave their written and informed consent to participate in the study. The study protocol and comprehensive characteristics of the study and control group have been described in detail elsewhere [13].

### Data collection

Study participants completed a paper survey questionnaire when they visited the outpatient clinic (control group) or rheumatology department (study group). The study used standardised survey instruments: The Inventory for the Measurement of Coping Strategies with Stress (Mini-COPE) and the Perceived Stress Scale (PSS-10). Both of these instruments have a Polish adaptation and validation [14]. The Mini-COPE assesses the strategies respondents use when stressful situations arise, while the PSS-10 assesses the intensity of stress related to one's life situation over the past month. A self-administered questionnaire was used to collect socio-demographic, treatment, and stress data.

Next, a blood sample was used to determine plasma level of cortisol and CRP. Blood was collected into vacuum test tubes in a volume of 5 ml from the ulnar vein. Between 7.45 and 8.15 a.m., venous blood was drawn. All assays were performed by immunoenzymatic ELISA. In RA patients, plasma levels of cortisol and CRP were determined during routine blood draws. Rules were adopted regarding the method, patient preparation, and time associated with blood collection. Data on the DAS28 questionnaire evaluating disease activity by a rheumatologist were obtained from the medical records.

### Ethical considerations

The study was conducted according to the guidelines of the Declaration of Helsinki and has been approved by the University Bioethics Committee (protocol code 122.6120.293.2016, approval date 28/04/2017). All respondents were informed about the goal of the project, and each of them provided an informed consent.

### Statistical methods

The mean, standard deviation, median, and minimum and maximum values were used to describe quantitative variables. The conformity of these variables’ distributions with the normal distribution was tested using the Shapiro–Wilk test. The control and study groups were compared using the Mann–Whitney *U* test. Differences between the control and study groups (in terms of socio-demographic characteristics) were assessed using the Pearson’s chi-square test or Fisher’s exact test (data not shown). To assess the correlation between (1) cortisol level, (2) plasma CRP protein levels, and (3) the scores of the DAS28, and Mini-COPE or PSS-10 questionnaire scores, Spearman’s Rank-Order Correlation was applied. Analyses were performed using IBM SPSS Statistic ver. 28 for Windows (SPSS Inc., Chicago, IL, USA). The accepted level of statistical significance was 0.05.

## Results

A total of 164 participants took part in the study, of whom 84 had RA, while the remaining 81 were the control group. Almost 80% were women. The largest group was between 50 and 59 (30.3%) and 60–70 years old (37.6%). The study and control groups were comparable, with the only significant differences observed in employment status. Those in the control group were predominantly economically active (71.6%), while the study group had the highest number of people on a pension (53.6%).

The largest group of patients with RA, 58%, was those with the disease for more than 10 years. 16% of the respondents had been ill for 1–5 years and 11% of the patients had been ill for 6–9 years. The remainder had been ill for less than 1 year.

All patients remained in treatment; of these, only a third of patients received biological treatment, while 59.5% were treated with glucocorticosteroids. Nearly 80% of the respondents believed that stress contributed to the onset of the disease and almost 90% of the patients were convinced that stress influenced the course of the disease.

The mean value of the perceived stress intensity measured on the PSS-10 scale was not statistically significantly different between the respondents in the control and study groups (20.0 vs. 20.1, *p = *0.889).

When analysing stress coping strategies, it can be observed that among all participants (RA and combined control group combined), the most frequently used coping strategies were *active coping* (2.2), *planning* (2.2), and *acceptance* (2.0). In contrast, respondents were least likely to use the following coping methods to deal with stress: *use of psychoactive substances* (0.2), *cessation of activities* (0.8), *denial* (0.8), and *sense of humour* (0.8). The results for the Mini-COPE scale were very similar between the comparison groups, with a few exceptions. Patients with RA compared to the control group were significantly more likely to use the strategy of *turning to religion* (1.8 vs. 1.4; *p = *0.012). On the other hand, the control group was characterised by a more frequent use of the *sense of humour* strategy than the study group (0.9 vs. 0.6; *p = *0.003) (Table 1).

**Table 1:** Stress coping strategies among study participants (*n = *165)

Strategies for coping with stress Mini-COPE	All respondents (*n = *165)	Study group (RA) (*n = *84)	Control group (*n = *81)	*p*^a^
Active coping				
Mean	2.2	2.2	2.3	0.352
Standard deviation	0.6	0.6	0.6	
Median	2.0	2.0	2.0	
Minimum	0.5	1.0	0.5	
Maximum	3.0	3.0	3.0	
Planning				
Mean	2.2	2.1	2.2	0.129
Standard deviation	0.6	0.7	0.6	
Median	2.0	2.0	2.0	
Minimum	0	0.0	0.5	
Maximum	3.0	3.0	3.0	
Positive re-evaluation				
Mean	1.7	1.7	1.8	0.458
Standard deviation	0.6	0.6	0.7	
Median	2.0	1.8	2.0	
Minimum	0.0	0.0	0.0	
Maximum	3.0	3.0	3.0	
Acceptance				
Mean	2.0	2.1	2.0	0.536
Standard deviation	0.6	0.6	0.6	
Median	2.0	2.0	2.0	
Minimum	0.0	0.0	0.5	
Maximum	3.0	3.0	3.0	
Sense of humour				
Mean	0.8	0.6	0.9	0.003
Standard deviation	0.6	0.6	0.6	
Median	1.0	0.5	1.0	
Minimum	0.0	0.0	0.0	
Maximum	2.5	2.5	2.5	
Turning to religion				
Mean	1.6	1.8	1.4	0.012
Standard deviation	1.1	1.0	1.1	
Median	2.0	2.0	1.0	
Minimum	0.0	0.0	0.0	
Maximum	3.0	3.0	3.0	
Seeking emotional support				
Mean	1.9	1.9	1.9	0.731
Standard deviation	0.7	0.7	0.7	
Median	2.0	2.0	2.0	
Minimum	0.0	0.5	0.0	
Maximum	3.0	3.0	3.0	
Seeking instrumental support				
Mean	1.9	1.8	1.9	0.696
Standard deviation	0.7	0.8	0.6	
Median	2.0	2.0	2.0	
Minimum	0.0	0.0	0.0	
Maximum	3.0	3.0	3.0	
Dealing with something else				
Mean	1.9	1.9	1.9	0.559
Standard deviation	0.7	0.7	0.7	
Median	2.0	2.0	2.0	
Minimum	0.0	0.0	0.0	
Maximum	3.0	3.0	3.0	
Denial				
Mean	0.8	0.8	0.7	0.319
Standard deviation	0.8	0.8	0.7	
Median	0.5	0.5	0.5	
Minimum	0.0	0.0	0.0	
Maximum	3.0	3.0	2.5	
Discharge				
Mean	1.4	1.4	1.4	0.754
Standard deviation	0.7	0.7	0.6	
Median	1.5	1.5	1.5	
Minimum	0.0	0.0	0.0	
Maximum	3.0	3.0	2.5	
Use of psychoactive substances				
Mean	0.2	0.1	0.3	0.093
Standard deviation	0.5	0.4	0.5	
Median	0.0	0.0	0.0	
Minimum	0.0	0.0	0.0	
Maximum	2.5	2.0	2.5	
Cessation of activities				
Mean	0.8	0.9	0.7	0.076
Standard deviation	0.7	0.7	0.6	
Median	0.5	1.0	0.5	
Minimum	0.0	0.0	0.0	
Maximum	3.0	3.0	2.0	
Blaming yourself				
Mean	1.3	1.2	1.4	0.172
Standard deviation	0.7	0.7	0.6	
Median	1.0	1.0	1.5	
Minimum	0.0	0.0	0.0	
Maximum	3.0	3.0	2.5	

*Mini-COPE* Inventory for the measurement of coping with stress

^a^*p* for the Mann–Whitney *U* test comparing the distribution of variables between the control and study groups

An analysis was carried out on the relationship between cortisol levels and stress coping in patients with RA who were not treated with glucocorticosteroids (*n = *34). The results are presented in Table 2. It was observed that higher plasma cortisol concentrations occurred with a higher frequency of the following stress coping strategies: positive re-evaluation, *sense of humour, turning to religion,* and *seeking instrumental support*. However, it should be noted that these results were not statistically significant in the study group (women and men combined). In contrast, in the women's group, statistically significant correlations were found between higher cortisol levels and more frequent use of the following strategies: *positive re-evaluation* (rho = 0.515, *p = *0.014), *seeking emotional support* (rho = 0.439, *p = *0.041) and *seeking instrumental support* (rho =  0.476, *p = *0.025), as well as the *denial* strategy (rho = 0.471, *p = *0.027) (Table 2).

**Table 2:** Relationship between cortisol level and PSS-10 and Mini-COPE questionnaire scores—RA patients without glucocorticosteroid treatment (*n = *34)

Cortisol [ng/mL]	Total (*n = *34)		Women (*n = *22)		Men (*n = *12)	
	rho^a^	*p*^b^	rho	*p*	rho	*p*
PSS-10	− 0.125	0.482	− 0.100	0.657	− 0.190	0.555
Mini-COPE						
Active coping	0.135	0.447	0.300	0.174	− 0.363	0.247
Planning	0.005	0.978	0.110	0.625	− 0.306	0.333
Positive re-evaluation	0.295	0.090	0.515	0.014	− 0.199	0.535
Acceptance	− 0.024	0.892	− 0.011	0.960	− 0.061	0.850
Sense of humour	0.298	0.086	0.219	0.326	0.470	0.123
Turning to religion	0.328	0.058	0.302	0.171	0.306	0.333
Seeking emotional support	0.231	0.189	0.439	0.041	− 0.273	0.391
Seeking instrumental support	0.333	0.055	0.476	0.025	0.025	0.938
Dealing with something else	0.243	0.166	0.257	0.248	0.337	0.284
Denial	0.287	0.100	0.471	0.027	− 0.136	0.674
Discharge	0.094	0.597	− 0.029	0.899	0.380	0.223
Use of psychoactive substances	− 0.172	0.332	− 0.082	0.716	− 0.426	0.167
Cessation of activities	− 0.047	0.793	0.069	0.761	− 0.337	0.284
Blaming oneself	0.094	0.595	0.014	0.952	0.273	0.391

*PSS-10* Perceived stress scale, *Mini-COPE* Inventory for the measurement of coping with stress

^a^Spearman correlation coefficient

^b^*p* for Spearman’s rank-order correlation

The study also examined the relationship between plasma CRP protein levels and stress intensity. It could be observed that patients with high stress intensity also had higher CRP protein concentrations (10.0 ± 12.4 mg/L vs. 8.8 ± 12.0 mg/L; *p = *0.590). This association was statistically significant in the male group, where patients with high stress intensity had more than double the concentration of CRP protein compared to patients with lower stress intensity (19.4 ± 14.8 mg/L vs. 7.1 ± 5.7 mg/L; *p = *0.038) (Table 3).

**Table 3:** Plasma level of CRP protein and stress intensity as measured by the PSS-10 questionnaire among total RA patients and by gender (*n = *84)

CRP [mg/L]^a^	Patients with RA	Women	Men
Low and medium stress levels	*n = *38	*n = *27	*n = *11
Mean	8.8	9.5	7.1
SD	12.0	13.8	5.7
Median	6.1	5.9	7.2
Minimum	0.2	0.2	0.7
Maximum	56.0	56.2	16.0
High stress levels	*n = *46	*n = *36	*n = *10
Mean	10.0	7.3	19.4
SD	12.4	10.4	14.8
Median	5.3	3.7	15.2
Minimum	0.1	0.1	4.5
Maximum	52.5	52.5	49.6
*p*^b^	0.590	0.994	0.038

*PSS-10* Perceived stress scale, *SD* Standard deviation

^a^C-reactive protein

^b^*p* for Mann–Whitney *U* test to assess the association between CRP protein levels and PSS-10

The relationship between CRP protein levels and the type of stress coping strategies used was investigated. It was observed that as CRP protein levels increased, patients were more likely to use a denial strategy. This association was found to be significant for the whole group of patients (rho = 0.283, *p = *0.009) and in the group of women (rho = 0.358, *p = *0.004). In women, there was also a negative correlation between CRP protein levels and the psychoactive substance use strategy, which meant that the higher the concentration of this protein in the blood, the less psychoactive substances were used (Table 4).

**Table 4:** Relationship between plasma CRP protein level and Mini-COPE and PSS-10 questionnaire scores among total RA patients and by gender (*n = *84)

CRP [mg/L]	Patients with RA *n = *84		Women *n = *63		Men *n = *21	
	rho^a^	*p*^b^	rho	*p*	rho	*p*
PSS-10	0.063	0.570	0.012	0.927	0.399	0.073
Mini-COPE						
Active coping	− 0.024	0.827	− 0.002	0.987	− 0.355	0.114
Planning	− 0.097	0.383	− 0.094	0.468	− 0.266	0.243
Positive re-evaluation	− 0.037	0.736	0.012	0.926	− 0.112	0.629
Acceptance	− 0.031	0.781	− 0.054	0.676	− 0.032	0.890
Sense of humour	0.067	0.547	0.029	0.820	− 0.034	0.883
Turning to religion	0.040	0.719	0.015	0.906	0.175	0.449
Seeking emotional support	0.095	0.394	0.235	0.066	− 0.259	0.257
Seeking instrumental support	− 0.30	0.787	0.018	0.889	− 0.027	0.909
Dealing with something else	− 0.068	0.541	− 0.119	0.357	0.176	0.445
Denial	0.283	0.009	0.358	0.004	0.144	0.532
Discharge	0.019	0.862	− 0.008	0.951	0.256	0.263
Use of psychoactive substances	− 0.075	0.502	− 0.333	0.008	0.075	0.746
Cessation of activities	− 0.089	0.426	− 0.051	0.693	− 0.051	0.828
Blaming oneself	0.016	0.886	0.049	0.706	− 0.185	0.423

*PSS-10* Perceived stress scale, *Mini-COPE* Inventory for the measurement of coping with stress, *CRP* C-reactive protein

^a^Spearman correlation coefficient

^b^*p* for Spearman’s rank-order correlation

The results of the analyses on the value of the association between the DAS28 index and the intensity of stress as measured by the PSS-10 questionnaire show that in patients with RA, as well as by sex. Patients demonstrating a high intensity of perceived stress were found to have a slightly higher DAS28 index, indicating greater exacerbation of the disease. On average, those with low-to-moderate perceived stress scored 3.6 ± 1.2 points on the DAS28 scale and those with high perceived stress scored 3.9 ± 1.3 points. Similar differences in DAS28 could be observed in the group of women with low or high stress intensity. The DAS28 in men with low stress intensity averaged 3.9 ± 1.0 points, while high stress correlated with a higher DAS28, averaging 4.6 ± 1.3 points.

The results of the analysis of the association between the DAS28 index and the type of stress coping strategies used show that as the value of the DAS28 index increased, patients were more likely to use a denial strategy. This association was significant for the entire group (rho = 0.301, *p = *0.005) and in the female group (rho = 0.355, *p = *0.004). In women, it was also found that a higher DAS28 index meant less frequent use of the strategy of using psychoactive substances (rho = − 0.335, *p = *0.007). On the contrary, among men, higher DAS28 was correlated with a less frequent use of active coping strategies (rho = − 0.396, *p = *0.076) and a more frequent use of strategies that involve dealing with something else (rho = 0.389, *p = *0.082) and discharge (rho = 0.420, *p = *0.058) (Table 5).

**Table 5:** Relationship between the scores of the DAS28 and Mini-COPE and PSS-10 questionnaire among total RA patients and by gender (*n = *84)

DAS28	Patients with RA *n = *84		Women *n = *63		Men *n = *21	
	rho^a^	*p*^b^	rho	*p*	rho	*p*
PSS-10	0.070	0.527	0.022	0.862	0.229	0.319
Mini-COPE						
Active coping	− 0.053	0.629	0.010	0.936	− 0.396	0.076
Planning	− 0.086	0.435	− 0.038	0.767	− 0.338	0.134
Positive re-evaluation	− 0.071	0.519	− 0.041	0.748	− 0.029	0.900
Acceptance	− 0.049	0.661	− 0.060	0.642	− 0.006	0.978
Sense of humour	0.066	0.553	− 0.005	0.969	0.192	0.403
Turning to religion	0.043	0.699	0.002	0.985	0.144	0.533
Seeking emotional support	0.133	0.228	0.233	0.066	− 0.046	0.843
Seeking instrumental support	0.043	0.699	0.089	0.487	0.054	0.815
Dealing with something else	− 0.018	0.868	− 0.119	0.352	0.389	0.082
Denial	0.301	0.005	0.355	0.004	0.132	0.570
Discharge	0.065	0.555	0.007	0.954	0.420	0.058
Use of psychoactive substances	− 0.107	0.332	− 0.335	0.007	0.084	0.717
Cessation of activities	− 0.067	0.543	− 0.056	0.662	0.055	0.814
Blaming oneself	0.107	0.334	0.119	0.353	0.023	0.922

*PSS-10* Perceived stress scale, *Mini-COPE* Inventory for the measurement of coping with stress, *DAS28* Disease activity score

^a^Spearman correlation coefficient

^b^*p* for Spearman’s rank-order correlation

## Discussion

The issue of stress and how to cope with it is one of the most important issues in modern psychology. Stress can mobilise the body to fight, but it also contributes to psychological discomfort, adjustment difficulties, and is very likely to initiate the onset and development of many diseases. The very occurrence of a chronic disease becomes a source of severe psychological stress for the patient. Many observations indicate that stress not only influences the onset of illness, but also affects patient behaviour and course of treatment [15].

Rheumatoid arthritis is a chronic disease that significantly changes the lifestyle of patients. Pain accompanying the disease, increasing disability, alternating periods of remission, and exacerbation of the disease affect many spheres of life, and cause stressful situations, and consequently emotional disorders in patients with RA [16, 17].

RA patients very often develop psychiatric and anxiety disorders, most often due to changes in the body during the course of the disease, chronic pain, difficulties in daily life, emotional burden, and the unpredictability of RA. The disorder can also be caused by organic changes in the nervous system that result from the disease. Depression occurs in 13 to 65% of patients with RA. It is assumed that an intense production of pro-inflammatory cytokines, including interleukins IL-6, IL-17, and TNF-α, may be responsible for this occurrence. Increased concentrations of interleukins contribute to reduced production of the neurotransmitter serotonin and consequently to decreased mood and the appearance of depressive symptoms. Untreated psychiatric disorders are associated with increased disease symptoms and worsen the patient's overall condition [18, 19].

In the era of today’s research, it is becoming extremely important to pay attention to the problems of the mental state of patients with RA. The 2015 EULAR recommendations on patient education with arthritis include evidence-based guidelines relating to self-medication, cognitive–behavioural therapy, and stress management. RA as a chronic disease forces a change in the lifestyle of patients, making it a difficult psychological and existential experience for them. Consequently, the disease not only affects emotions and behaviour, but also initiates the occurrence of stressful life situations. Both the physical and psychological effects of the disease adversely affect patients’ compliance with treatment and, consequently, make it more difficult for them to recover [20].

When analysing stress in patients with RA, it can be said that a so-called vicious circle mechanism takes place here. The occurrence of stressful situations induces the appearance of disease symptoms and leads to an exacerbation of the disease. On the other hand, it is the occurrence of the disease that is a significant source of stress and contributes to an increased number of stressful situations. Knowledge of stress coping strategies in this group of patients is fundamentally important to improve the quality of medical and psychological care, and the ability to cope with stress helps patients better adapt to life with the disease and supports the treatment process [21].

The presented research showed that almost 80% of the respondents believed that stress contributed to their illness and almost 90% of the patients were convinced that stress influenced the course of their illness.

Stress coping strategies are part of the adaptive process and refer to the cognitive and behavioural efforts that a person develops in relation to specific demands of oneself or the environment. These efforts are judged to be stressful or beyond the individual's capacity. Strategies that are taken in a specific stressful situation aim to change the situation or, if this is not possible, to mitigate its effects. The right way to cope with stress is of particular importance in chronically ill patients. Reduced stress through the use of appropriate coping strategies in the presence of illness helps patients adapt to the health situation at hand [4, 22].

The most commonly used stress coping strategies among RA patients were active coping, planning, and acceptance. Patients with RA were more likely to use the strategy of turning to religion compared to the control group. Janiszewska et al. [23] found slightly different results in their study in a group of RA patients. These respondents used the most active coping strategies, seeking instrumental support and positive re-evaluation. However, these strategies can be classified as positive strategies that promote adaptation to stressful situations. Tosato et al. [24] suggest that stress coping strategies can be an important factor influencing the perceived severity of illness and may become a useful tool to implement psychological interventions aimed at modifying specific coping styles.

When examining the relationship between CRP concentration and stress intensity, it was found that patients with RA characterised by high stress intensity also had higher CRP protein concentrations. On the other hand, when analysing the relationship between the concentration of CRP protein and the type of strategies used, it was observed that, as the CRP concentration increased, people with RA were more likely to use a denial strategy. In the presented study looking for a relationship between the DAS28 index and the intensity of stress, it was observed that patients with RA showing high stress intensity had an elevated DAS28 index. In their study on the prevalence of stress, anxiety symptoms, and depression in RA patients, Ruhaila and Chong showed that stress, depression, and anxiety were positively correlated with disease activity, functional status, number of tender joints, and overall health score [25].

As well as researchers Ng KJ et al. in their publication confirm that patients exposed to anxiety and depression have increased disease activity as determined by the DAS28 index [26]. When examining the relationship between stress coping strategies and the DAS28 index, they found that as the DAS28 index increased, the patients with RA were more likely to use a denial strategy. Among female RA patients, an increase in the DAS28 index was associated with more frequent use of the emotional support, whereas in male RA patients, a higher value of the DAS28 index value was associated with less frequent use of active coping strategies and more frequent use of strategies of negation and discharge.

The interdependence between psychological and physical processes in patients with RA requires an understanding of the multiple levels at which this interaction can occur. Adapting patients to disease-related stressful situations through the skillful use of coping strategies can have short- and long-term consequences for these patients. A holistic approach that takes into account not only physical and laboratory findings, but also psychosocial factors that interact with the patient can result in improved treatment efficacy and slower disease activity.

## Conclusion

The study confirmed that there are associations between the intensity of stress and the coping strategies and the concentration of CRP protein and the index of disease activity. However, the work has its advantages and disadvantages. The advantages include the fact that an attempt was made to analyze the relationship between stress intensity, stress coping strategies, and the clinical status of patients with rheumatoid arthritis using a control group. The results deepen the knowledge about the strategies RA patients use to cope with stressful situations and how the ability to cope with stress affects their clinical status. The results may contribute to the implementation of behavioural therapies as an adjunct to drug treatment. The limitations of the study include the use of a convenience sample rather than a random sample, the study in a limited area, and the size of the study group, which does not allow for the generalisation of the findings to the entire population of RA patients. There is a need to further explore the problem of stress and coping strategies in patients diagnosed with RA to quickly implement helpful solutions and prevent the negative effects of stress.

## Data Availability

The corresponding datasets of this study are available from the corresponding author on reasonable request.
